# Line it up—Inadvertent placement of nasogastric tube in pleural space resulting in iatrogenic empyema

**DOI:** 10.1002/ccr3.4729

**Published:** 2021-08-30

**Authors:** Murtaza S. Hussain, Smit S. Deliwala, Dominic Awuah, Aashish Valvani

**Affiliations:** ^1^ Department of Internal Medicine Michigan State University at Hurley Medical Center Flint Michigan USA; ^2^ Division of Pulmonary/Critical Care Department of Internal Medicine Michigan State University at Hurley Medical Center Flint Michigan USA

**Keywords:** feeding tube, malposition, nasogastric tube, perforation

## Abstract

Dobhoff tubes, used for post‐pyloric feedings, have a weighted metal end with a small diameter that enhances their flexibility to traverse the gastrointestinal tract. Unfortunately, the metal stylet can iatrogenically perforate surrounding structures in patients with diminished cough and gag (1), and extreme caution should be considered before its utilization.

## Graphical abstract

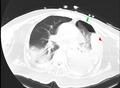

## 1 CASE HISTORY

Dobhoff tubes, used for post‐pyloric feedings, have a weighted metal end with a small diameter that enhances their flexibility to traverse the gastrointestinal tract. Unfortunately, the metal stylet can iatrogenically perforate surrounding structures in patients with diminished cough and gag,^1^ and extreme caution should be considered before its utilization.

Enteral nutrition access is classified as gastric and post‐pyloric. Gastric feedings, such as orogastric and nasogastric tube, are used in lower risk patients. Post‐pyloric feedings are utilized in critically ill patients to mitigate aspiration. As with other feeding modes, Dobhoff tubes require a clinical and radiologic confirmation for proper placement.

### 1.1 Question

What are the implications of incorrect Dobhoff tube placement?

### 1.2 Answer

Dobhoff tubes can easily perforate vital surrounding structures and placement should be radiologically confirmed; not just clinically with auscultation of the epigastrium.

**FIGURE 1:**
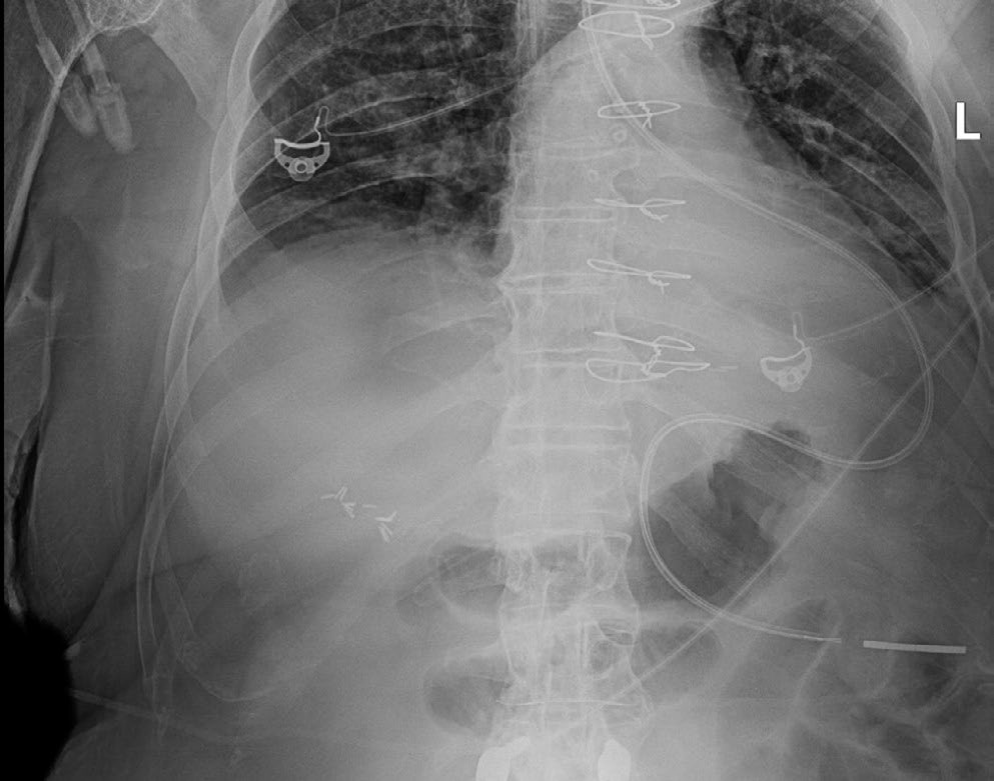
Abdominal radiograph after Dobhoff tube placement. It shows a far lateral trajectory; going up in the left lower lobe, piercing the pleura and diaphragm, and the metal tip is seen below the diaphragm

**FIGURE 2:**
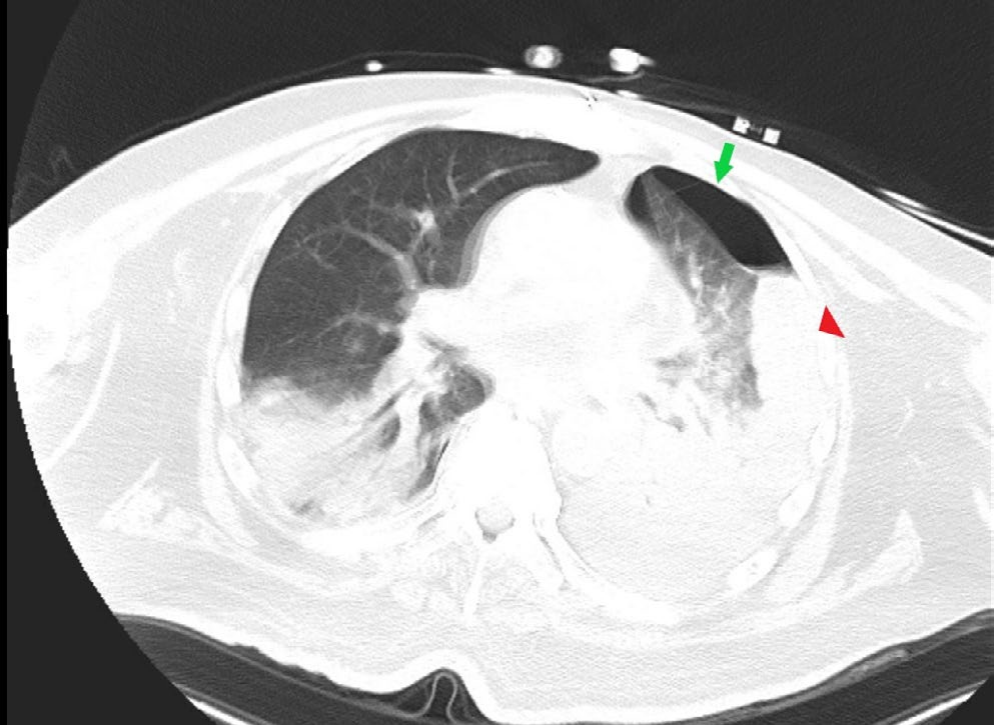
Computed tomography (CT) axial view of lungs the morning after feeding. Small pneumothorax seen with green arrow, and iatrogenic hydrothorax highlighted with red arrowhead

A 66‐year‐old male patient from a nursing facility presented with encephalopathy from septic shock and required a prolonged stay in the intensive care unit. A nasogastric tube was placed and visually confirmed to be under the diaphragm on consecutive abdominal radiographs (Figure 1); tube feeding was subsequently initiated. Within a few hours, the patient started desaturating. An emergent computed tomography (CT) of the chest and abdomen (Figure 2) revealed a new left hydropneumothorax ^2^ and pleural effusion from iatrogenic puncture of pleura. Tube feedings were stopped, and a chest tube was placed immediately with drainage and eventual seal. However, the overall prognosis was poor and patient's wife eventually agreed to comfort care; he passed 1 day later.

## CONFLICT OF INTEREST

None declared.

## AUTHOR CONTRIBUTIONS

Murtaza S. Hussain involved in acquisition, draft, and review. Smit S. Deliwala involved in conception, draft, and review. Dominic Awuah involved in acquisition and review. Aashish Valvani involved in review.

## ETHICAL APPROVAL

Appropriate informed consent was obtained from patient and his wife for possible publication of image. His wife verbalized understanding.

## Data Availability

Data sharing is not applicable to this article as no new data were created or analyzed in this study.

## References

[1] Lo JO, Wu V, Reh D, Nadig S, Wax MK. Diagnosis and management of a misplaced nasogastric tube into the pulmonary pleura. Arch Otolaryngol Head Neck Surg. 2008;134(5):547‐550. 18490579 10.1001/archotol.134.5.547

[2] Abidali A, Mangram A, Shirah GR, et al. Bilateral pneumothoraces in a trauma patient after dobhoff tube insertion. Am J Case Rep. 2018;19:244. 29503437 10.12659/AJCR.906846 PMC5850843

